# GUCY2C lysosomotropic endocytosis delivers immunotoxin therapy to metastatic colorectal cancer

**DOI:** 10.18632/oncotarget.2455

**Published:** 2014-09-08

**Authors:** Glen P. Marszalowicz, Adam E. Snook, Michael S. Magee, Dante Merlino, D. Berman-Booty Lisa, Scott A. Waldman

**Affiliations:** ^1^ School of Biomedical Engineering, Science, and Health Systems, Drexel University, Philadelphia, PA, USA; ^2^ Department of Pharmacology and Experimental Therapeutics, Thomas Jefferson University, Philadelphia, PA, USA; ^3^ Cancer Biology, Thomas Jefferson University, Philadelphia, PA, USA

**Keywords:** GUCY2C, immunotoxin, therapeutic targeting, metastatic colorectal cancer

## Abstract

The emergence of targeted cancer therapy has been limited by the paucity of determinants which are tumor-specific and generally associated with disease, and have cell dynamics which effectively deploy cytotoxic payloads. Guanylyl cyclase C (GUCY2C) may be ideal for targeting because it is normally expressed only in insulated barrier compartments, including intestine and brain, but over-expressed by systemic metastatic colorectal tumors. Here, we reveal that GUCY2C rapidly internalizes from the cell surface to lysosomes in intestinal and colorectal cancer cells. Endocytosis is independent of ligand binding and receptor activation, and is mediated by clathrin. This mechanism suggests a design for immunotoxins comprising a GUCY2C-directed monoclonal antibody conjugated through a reducible disulfide linkage to ricin A chain, which is activated to a potent cytotoxin in lysosomes. Indeed, this immunotoxin specifically killed GUCY2C-expressing colorectal cancer cells in a lysosomal- and clathrin-dependent fashion. Moreover, this immunotoxin reduced pulmonary tumors >80% (p<0.001), and improved survival 25% (p<0.001), in mice with established colorectal cancer metastases. Further, therapeutic efficacy was achieved without histologic evidence of toxicity in normal tissues. These observations support GUCY2C-targeted immunotoxins as novel therapeutics for metastatic tumors originating in the GI tract, including colorectum, stomach, esophagus, and pancreas.

## INTRODUCTION

Immunotoxins (ITs) have emerged as a key weapon in the arsenal against metastatic cancer [1, 2]. Originally described by Ehrlich, ITs are the embodiment of his concept of the magic bullet, combining the specific targeting of antibodies with the exquisite potency of cytotoxins to kill cells [3, 4]. Early studies combining holotoxins and antibodies against proteins with increased expression in cancer failed because of the absence of tumor specificity, producing normal tissue destruction [1, 2]. Improvements in natural toxin payloads have removed their promiscuous binding domains, emulated their linkages to these binding domains, and modified antigenic determinants to evade immune clearance [5]. In addition, novel cytotoxic payloads are emerging, including semiconductor quantum dots for photodynamic therapy and nanoshells which encapsulate traditional drugs but cloak them from normal tissue [6]. However, while cytotoxic payloads and linker technologies have evolved, one limitation to clinical implementation of immunotoxins is the availability of tumor-specific targets with cellular dynamics that effectively deploy cytotoxic payloads.

Guanylyl cyclase C (GUCY2C), the cell surface receptor for diarrheagenic bacterial heat-stable enterotoxins and the endogenous paracrine hormones guanylin and uroguanylin [7], is primarily expressed in apical membranes [8] of intestinal epithelial cells [9-12], segregated from the systemic compartment by the intestinal barrier [13-18]. Similarly, it is expressed by select neurons in hypothalamus, mediating a novel gut brain endocrine axis regulating appetite [19], and separated from the systemic compartment by the blood-brain barrier [20]. Importantly, GUCY2C is over-expressed by primary and metastatic colorectal tumors [9-11, 21, 22]. In that context, GUCY2C has emerged as a novel vaccine target to treat and prevent colorectal cancer metastases without normal tissue damage [23-26]. These characteristics suggest that GCC may qualify as an effective therapeutic target for immunotoxins in metastatic colorectal cancer, the second leading cause of cancer mortality in the United States and the fourth most common cause of cancer worldwide [27]. Here, we define the cellular dynamics of GUCY2C, and leverage those characteristics to develop immunotoxins with therapeutic utility in mouse models of metastatic colorectal cancer.

## RESULTS

### mAbs Recognize the Extracellular Domain, Without Disrupting Receptor Function, in GUCY2C

We created three unique mouse mAbs (MS7, MS20, MS24) [19] that specifically recognize the extracellular domain of GUCY2C (Supplemental Fig. 1A). MS20 recognized both mouse and human GUCY2C, and was utilized in the present studies. These mAbs did not directly activate GUCY2C and did not block its activation by ST (Supplemental Fig. 1B), demonstrating their inactivity as pharmacophores at the receptor binding site and their utility as inert probes of GUCY2C cellular dynamics.

### Lysosomotropic Endocytosis of GUCY2C

**Figure 1:**
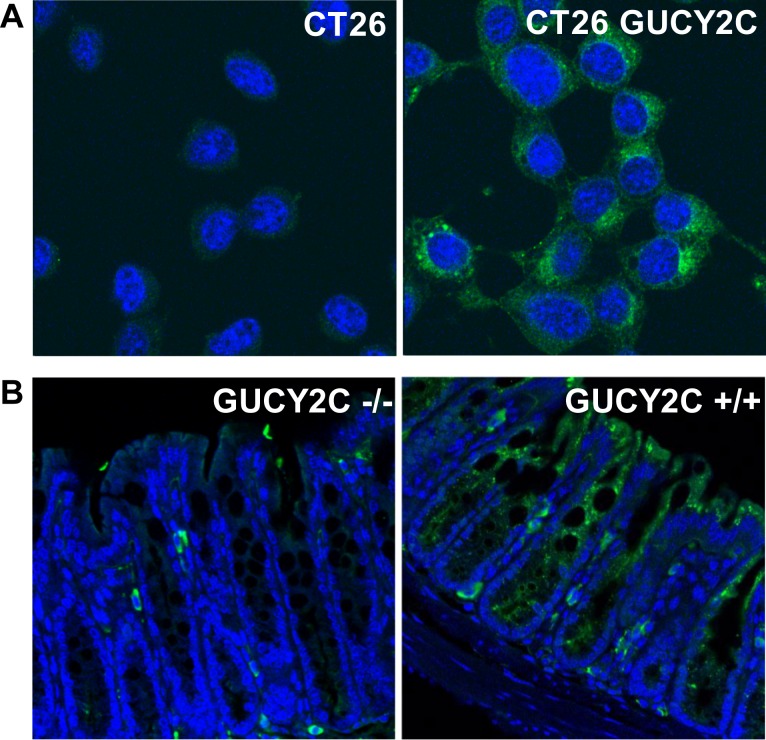
Intracellular localization of GUCY2C in intestinal epithelial and colorectal cancer cells Immunofluorescent staining with GUCY2CmAb (green), with DAPI counterstain for nuclei (blue), in (A) CT26 and CT26.GUCY2C murine colorectal cancer cells or (B) colon from GUCY2C KO (GUCY2C−/−) or WT (GUCY2C+/+) mice. Results are representative of at least three independent replicates.

**Figure 2:**
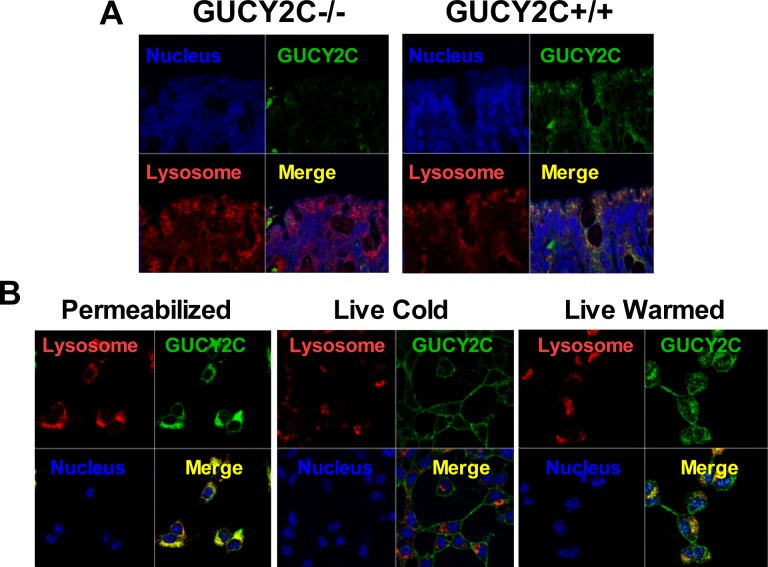
Lysosomotropic endocytosis of GUCY2C (A) GUCY2C immunofluorescence (green) co-localized (yellow) with lysosomes (red) in colons from GUCY2C+/+, but not GUCY2C−/−, mice (60x magnification). (B) CT26.GUCY2C cells imaged after methanol fixation (left), after 3 h exposure to GUCY2CmAb at 4°C in DMEM, washed with PBS and methanol-fixed (center), or after 3 h exposure to GUCY2CmAb at 4°C in DMEM, washed with PBS, warmed in DMEM at 37°C for 1 h and then methanol-fixed (right). Following methanol fixation and permeabilization, cells were imaged for GUCY2CmAb (green), lysosomes with antibodies to Lamp1 (red), and nuclei with DAPI (blue). Results are representative of at least three independent replicates.

CT26 mouse colorectal cancer cells [28], which are devoid of endogenous GUCY2C expression, were engineered to express mouse GUCY2C (CT26.GUCY2C) [23-25]. Unexpectedly, whole cell IF staining with MS20 revealed that GUCY2C primarily resided in the intracellular compartment (Fig. 1A) rather than on the cell surface as suggested by the prevailing paradigm [14, 29-32]. Similarly, GUCY2C in mouse intestine exhibited the same predominant subcellular distribution in wild type (GUCY2C^+/+^), but not in GUCY2C-deficient (GUCY2C^−/−^), mice (Fig. 1B). Co-staining with MS20 and Lamp1 demonstrated localization of GUCY2C within lysosomes of colonocytes in wild-type (GUCY2C^+/+^), but not GUCY2C-deficient (GUCY2C^−/−^), mice (Fig. 2A, Supplemental Video 1). Also, whole cell IF revealed GUCY2C localization within lysosomes in CT26.GUCY2C cells (Fig. 2B, left). Live cell staining at 4°C, which prevents endocytosis [33], demonstrated that MS20 was limited only to the cell surface of GUCY2C in CT26.GUCY2C cells (Fig. 2B, middle). However, warming to 37°C released MS20-GUCY2C complexes to internalize from the cell surface to lysosomes in the absence of canonical ligands (Fig. 2B, right; Supplemental Video 2). Indeed, MS20-GUCY2C complexes exhibited rapid ligand-independent internalization (Supplemental Fig. 2). Additionally, endogenously expressed GUCY2C internalized to lysosomes in STC1 murine intestinal cancer cells (Supplemental Fig. 3). These observations demonstrate that GUCY2C undergoes rapid ligand-independent internalization from the cell surface into the lysosomal compartment in intestinal epithelial and colorectal cancer cells.

### Lysosomotropic Endocytosis Requires Clathrin, But Not Caveolin or the GUCY2C Cytoplasmic Domain

**Figure 3:**
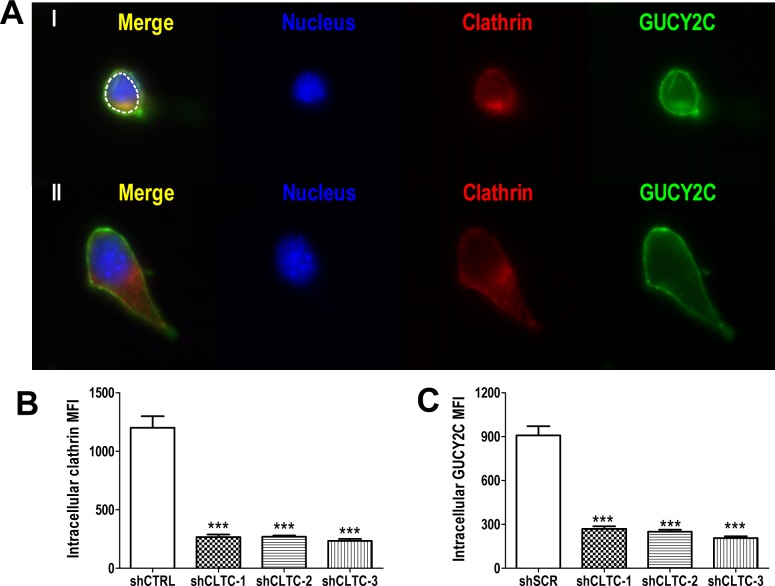
Lysosomotropic endocytosis of GUCY2C is clathrin-dependent (A) Representative single cell images showing internalized GUCY2C (green) detected as in A-C in cells with stably transfected control (I) or clathrin-specific (II) shRNA. Cells were counterstained with antibodies to clathrin (red) and DAPI for nuclei (blue). The intracellular compartment was selected for quantification (dotted white line). (B) Quantification of clathrin knockdown and (C) GUCY2C internalization in cells treated with control (shCTRL) or three different clathrin-specific shRNAs (shCLTC-1, 2 and 3). Analyses were performed in a blinded fashion and data are mean ± SEM of at least ten cells. *** p<0.0001, one-way ANOVA.

Receptor-dependent endocytosis is mediated by clathrin [34], and reducing expression of this scaffolding protein using three different targeted shRNA constructs proportionately reduced internalization of GUCY2C into lysosomes (Fig. 3A-C). Indeed, these analyses revealed that at least 65% of the complement of GUCY2C resided within the intracellular compartment (Fig. 3C). In contrast, reducing the expression of caveolin, which mediates clathrin-independent endocytosis [35], in CT26.GUCY2C cells did not alter GUCY2C lysosomotropic endocytosis (Supplemental Fig.4). Moreover, a truncation mutant of GUCY2C which lacks all intracellular regions and catalytic activity, but retains extracellular and transmembrane domains and the ability to bind GUCY2C ligands (GUCY2Ctm) [25], also internalized by a clathrin-dependent, caveolin-independent mechanism (Supplemental Fig. 5).

### Targeting GUCY2C Lysosomotropic Endocytosis with Immunotoxins

**Figure 4:**
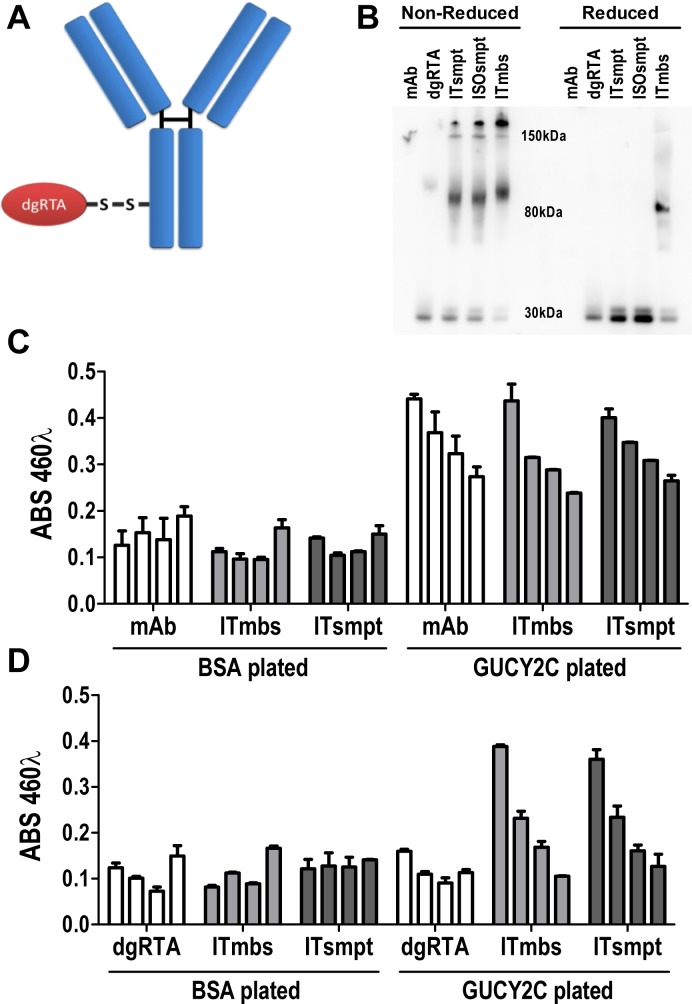
GUCY2C-targeted immunotoxin delivers dgRTA specifically to GUCY2C (A) The design of cleavable ITsmpt conjugates of dgRTA and GUCY2C-specific mAb. (B) Immunoblot detecting ricin A chain under non-reducing (left) or reducing (right) conditions. Unconjugated GUCY2CmAb (mAb) and dgRTA served as controls for ITsmpt (reducible), ISOsmpt, and ITmbs (non-reducible) conjugates. (C, D) ELISA titration of GUCY2CmAb, ITmbs or ITsmpt on plates coated with BSA or GUCY2C_1-430_, detected with (C) anti-mouse HRP or (D) anti-ricin/anti-rabbit HRP. Bars indicate means + SD of triplicate wells titrated from 0.01-10 μg/mL.

Internalization of GUCY2C-mAb complexes to lysosomes suggests that GUCY2C may be a particularly tractable target for delivery of ITs. We designed an IT in which the sterically-hindered cleavable disulfide linker 4-succinimidyloxycarbonyl-α-methyl-α-[2-pyridyldithio]-toluene (SMPT) [1, 2, 36] joined MS20 to dgRTA by the free sulfhydryl group created after reduction and removal of the ricin B chain (Fig. 4A) [37], resulting in an immunotoxin (ITsmpt) containing 1 dgRTA per IgG which can be liberated in lysosomes. As a negative control, dgRTA was conjugated to MS20 with m-maleimidobenzoyl-N-hydroxysuccinimide ester (MBS), producing an immunotoxin with a non-cleavable thioether bond (ITmbs) which cannot be liberated in lysosomes. Immunoblot analysis, probing for dgRTA under non-reducing conditions, revealed a 180 kDa conjugate consisting of the 30 kDa dgRTA and 150 kDa MS20 IgG (Fig. 4B). Slight reduction of immunoglobulin heavy and light chains during electrophoresis produced an 80 kDa species representing dgRTA conjugated to the heavy chain of IgG (Fig. 4B). Reduction released dgRTA from ITsmpt and isotype control IgG immunotoxin (negative control for the cleavable IT; ISOsmpt), but not ITmbs, producing 30 kDa (free dgRTA) and 80 kDa (dgRTA + IgG heavy chain) bands, respectively (Fig. 4B).

**Figure 5:**
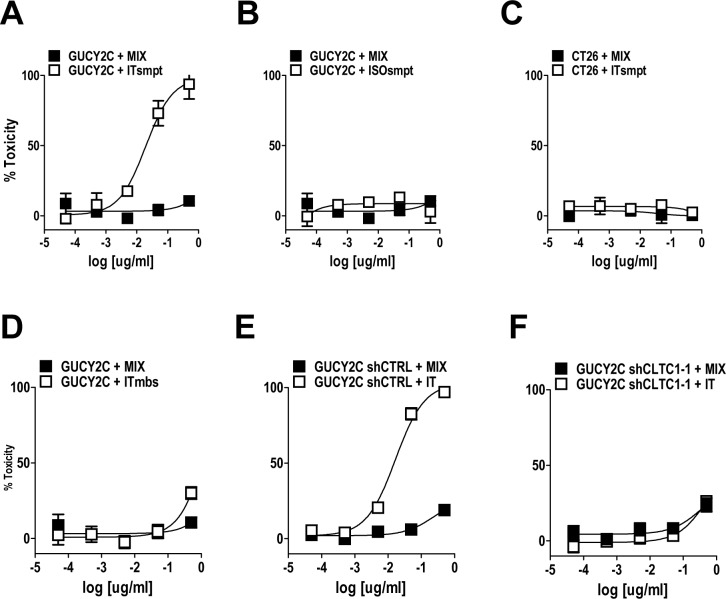
GUCY2C-targeted ITsmpt cytotoxicity depends on GUCY2C, lysosomal cleavage, and clathrin (A-D) CT26.GUCY2C cells were treated with ITsmpt (A) or the isotype control toxin conjugate ISOsmpt (B) for 48 h prior to MTT assay to quantify cytotoxicity. (C) Parental CT26 cells served as a negative control. (D) CT26.GUCY2C cells also were treated with the non-cleavable ITmbs. (E, F) The clathrin-dependence of ITsmpt cytotoxicity was quantified in CT26.GUCY2C cells treated with (E) control or (F) clathrin-specific shRNAs. All treatments (A-F) were compared to mixed, but not conjugated, molar equivalents of antibody + dgRTA (MIX). Results are the mean ± SEM of at least 3 independent determinations.

MS20 conjugated by cleavable or non-cleavable linkers recognized GUCY2C (Fig. 4C) and delivered antigen-targeted dgRTA (Fig. 4D). In that context, ITsmpt (Fig. 5A), but not ISOsmpt (Fig. 5B), specifically killed CT26.GUCY2C, but not CT26, cells (Fig. 5C) in a dose-dependent fashion. The non-cleavable ITmbs was minimally effective compared to ITsmpt (extrapolated Kd ITmbs, 1.158 μg/mL vs Kd ITsmpt, 0.019 μg/mL; p<0.0001; Fig. 5D), supporting liberation of dgRTA from the IT in lysosomes. Further, shRNA targeting clathrin eliminated the cytotoxicity of ITsmpt (Fig. 5E-F, Supplemental Fig. 6). Moreover, ITsmpt, but not ISOsmpt, specifically killed GUCY2Ctm in a clathrin-dependent fashion (Supplemental Fig. 6). As in CT26.GUCY2C cells, the non-cleavable ITmbs was only weakly active, compared to the cleavable ITsmpt, in CT26.GUCY2Ctm cells (extrapolated Kd ITmbs, 0.483 μg/ml vs Kd ITsmpt, 0.016 μg/mL; p<0.0001; Supplemental Fig. 6). Thus, MS20 IT targets antibody-drug conjugate to cell surface GUCY2C which mediates endocytosis of the complex in a ligand- and GUCY2C cytoplasmic domain-independent, but clathrin-dependent, fashion, delivering ITs to lysosomes where they are cleaved, liberating cytotoxic dgRTA.

### GUCY2C-Targeted IT Opposes Colorectal Cancer Metastases Without Injuring Normal Tissues

**Figure 6:**
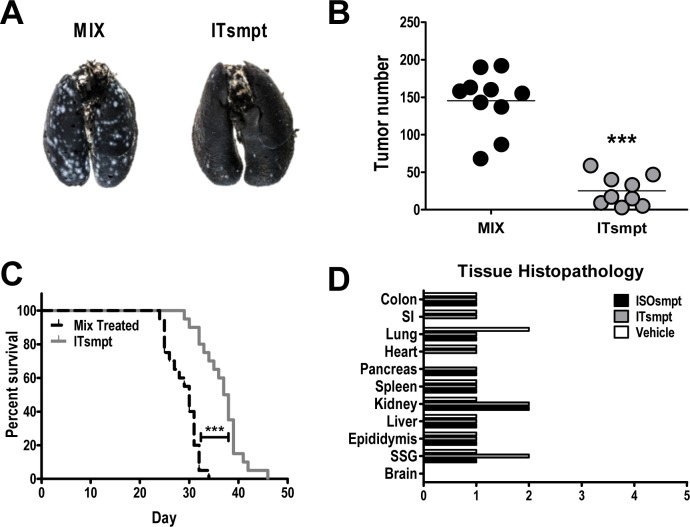
GUCY2C-targeted ITsmpt reduces colorectal cancer metastases to lung and increases survival in mice Mice were challenged IV with 5 × 10^5^ CT26.GUCY2C cells and treated with 0.4 mg/kg ITsmpt or MIX every other day beginning two days after tumor challenge. (A, B) For some mice, lungs were collected on day 14, (A) stained, and (B) tumors enumerated. (C) Remaining mice (at least 9 per cohort) were followed for survival. **** p<0.0001, one-way ANOVA. (D) Tissue toxicities in mice treated with 0.4 mg/kg ISOsmpt, 0.4 mg/kg ITsmpt, or vehicle control (3 mice per group). Histopathologic injury was scored as described in Materials and Methods.

ITsmpt administered by tail vein to mice bearing CT26.GUCY2C lung metastases in a regimen of 40 mg/kg every other day for 6 doses reduced tumor burden >80% (p<0.001) quantified after 12 days of therapy (Fig. 6A and B). In contrast, ITsmpt treatment of mice inoculated with parental CT26 cells was without effect on tumor metastases in lung (Sup. 7). Moreover, this regimen of ITsmpt increased survival 25% (p<0.001) in mice bearing CT26.GUCY2C lung metastases (Fig. 6C). Importantly, this regimen of ITsmpt was without specific adverse effects in normal tissues, including sites in which GUCY2C is normally expressed (intestine, brain; Fig. 6D). Indeed, thorough histopathologic examination of brain, colon, epididymus, heart, kidney, liver, lung, salivary gland, small intestine, and spleen did not reveal any evidence of significant clinical toxicity specifically produced by the GUCY2C-targeted IT (Fig. 6D).

## DISCUSSION

GUCY2C is uniquely suited as a therapeutic target for antibody-drug conjugates for metastatic disease. It is selectively expressed primarily by intestinal epithelia cells, from the duodenum to the rectum, where it is a key regulator of homeostatic processes organizing the crypt-surface axis [38-41]. Also, GUCY2C is expressed by select hypothalamic neurons, where it mediates a novel gut-neural axis regulating appetite and satiety [19]. These anatomical compartments serve as barriers, with tight segregation from the systemic circulation, structurally and functionally insulating them from circulating GUCY2C-targeted cells and macromolecules [9-12, 20]. Indeed, GUCY2C-targeted imaging agents in the circulation do not distribute to the gastrointestinal tract or central nervous system [10]. Moreover, GUCY2C-targeted vaccines generate systemic T cell and antibody responses without affecting the gastrointestinal tract or central nervous system [23-25]. Beyond this compartmentalization, GUCY2C is over-expressed on the surface of primary and metastatic colorectal tumors [9, 22, 42]. These characteristics, in which GUCY2C in normal tissues is compartmentalized and insulated outside the systemic compartment, but over-expressed on metastatic tumors residing within the systemic compartment, make it a highly specific functional tumor target to direct immunotoxins.

While anatomic compartmentalization coupled with general association with metastatic disease suggest unique utility as a therapeutic target, the cellular dynamics of GUCY2C, beyond its expression at the cell surface, has remained incompletely defined. The prevailing paradigm suggests that GUCY2C is primarily deployed in apical brush border membranes of intestinal epithelial cells, available to interact with its cognate paracrine ligands produced in the lumen of intestine [14, 29-32]. GUCY2C internalization has been quantified *in vitro* with radiolabeled ligand, although the intracellular fate of endocytosed complexes was unknown [43]. Similarly, imaging studies of tumors in mice using receptor-targeted ligands revealed accumulation of label inside tumor cells specifically mediated by GUCY2C although, again, the fate of internalized complexes remained undefined [10].

Here, development of monoclonal antibodies directed to the extracellular ligand-binding domain, but without effect on receptor activities (binding, catalytic activation), provided a unique opportunity to probe the fate of GUCY2C internalized from the cell surface. In striking contrast to the prevailing paradigm [14, 29-32], these analyses revealed that the majority of the GUCY2C associated with intestinal epithelial and colorectal cancer cells resided in the intracellular compartment, primarily in lysosomes. Live cell imaging directly visualized rapid internalization of GUCY2C from the cell surface into lysosomes. Like other guanylyl cyclases [44], endocytosis required clathrin, and disrupting its expression eliminated internalization of GUCY2C from the cell surface into lysosomes and the cytotoxic effects of GUCY2C-targeted ITs. Importantly, GUCY2C internalization was independent of ligands, and trafficking to lysosomes required the extracellular ligand binding, but not the cytoplasmic catalytic, domain, like other guanylyl cyclases [45, 46].

These previously unknown mechanistic elements of GUCY2C endocytosis inform a strategy to employ this receptor as a therapeutic target in metastatic colorectal cancer. Rapid internalization of GUCY2C independent of ligand binding, receptor activation or the cytoplasmic catalytic domain supports therapeutic targeting with inert structural probes of the extracellular domain, like antibodies. Similarly, endocytosis from the cell surface into lysosomes suggests that targeted agents can leverage the unique characteristics of these organelles and incorporate cytotoxins and linkers that optimize liberation to maximize cytotoxicity. In that context, dgRTA cannot access the cytoplasmic compartment of cells in the absence of an internalization partner, like an antibody, enhancing the specificity for targeted killing [1, 2, 37]. Further, dgRTA accesses ribosomal cytotoxic targets by activation in, and translocation from, lysosomes [1, 2, 37]. Moreover, the SMPT linker produces a sterically-hindered disulfide bond which resists disruption in the circulation, but maximizes release of dgRTA in the acidic pH and reductive environment of lysosomes [1, 2, 36].

Employing this mechanism-directed strategy, ITs targeted by MS20 and delivering the cytotoxic dgRTA, linked together with SMPT, killed mouse colorectal cancer cells *in vitro*. ITsmpt cytotoxicity was highly specific for GUCY2C, and isogenic colorectal cancer cells devoid of GUCY2C resisted cytotoxicity by this IT, while conjugates of non-specific immunoglobulin and dgRTA were inactive. Similarly, ITsmpt cytoxicity required internalization by antibody-GUCY2C complexes, and disrupting clathrin expression specifically eliminated IT efficacy, while mixtures of free MS20 and dgRTA were without effect. Moreover, IT cytotoxicity required activation in, and release from, lysosomes and MS20-dgRTA conjugates linked by the non-cleavable thioether MBS were 10- to 100-fold less potent compared to ITsmpt. Beyond cells *in vitro*, this mechanism-based approach translated into effective therapy in mice with established colorectal cancer metastases in lung. Indeed, six doses of ITsmpt administered every other day reduced metastatic disease >80%, and improved survival 25%, compared to mice receiving a mixture of free MS20 and dgRTA. Further, the effects of the targeted IT were highly specific for GCC-expressing tumors, and there were no obvious toxicities specifically associated with ITsmpt. In that context, tissues in which GUCY2C normally resides, including intestine and hypothalamus, were not affected by ITsmpt.

These studies reveal a novel biological mechanism mediating endocytosis of cell surface GUCY2C into lysosomes that is independent of receptor activity in normal intestinal epithelial and colorectal cancer cells. This mechanism can be leveraged to develop monoclonal antibody-directed ITs that specifically target GUCY2C-expressing colorectal cancer metastases, maximizing the efficacy of tumor cell cytoxicity while minimizing off target adverse effects in normal tissues, including extra-systemic compartments normally expressing GUCY2C. The emerging evolution in IT platforms, including humanizing monoclonal antibodies [47], novel cytotoxins like mayntansinoids [48], and next-generation scaffolds like pH-dependent boronate-linked linear-hyperbranched polymeric nanovehicles [49], underscores the importance of identifying therapeutic targets like GUCY2C that are tumor-specific, highly associated with disease, and mechanistically tractable to realize the full clinical potential of this therapeutic paradigm. The immediate translation of these approaches can best be appreciated by considering that beyond colorectal cancer, GUCY2C is ectopically expressed by a significant proportion of metastatic gastric, esophageal, and pancreatic tumors and GUCY2C-directed mAb ITs are in clinical development for their treatment [9, 50, 51].

## MATERIALS AND METHODS

### GUCY2C mAb Generation

The MS20 mAb was previously described [19]. MS7 and MS24 mAbs were similarly produced. All mAbs were purified with a protein G column (GE HiTrap Protein G HP, #17-0404-01).

### Cyclic GMP Accumulation

CT26 mouse colorectal cancer cells engineered to express GUCY2C (CT26.GUCY2C) [23-25], were grown to confluence in DMEM + 10% fetal bovine serum in a 12-well dish washed with three times with PBS, and pretreated with 500uL DMEM containing 1 mM 3-isobutyl-1-methylxanthine (IBMX) and 10 μg/mL antibody at 37°C for 30 min. Media was replaced with 500 μL fresh DMEM/IBMX/antibody mix containing 1 μM ST and incubated for an additional 30 min at 37°C prior to collection. ST is the heat-stable enterotoxin produced by enteroxigenic bacteria that cause Travelers' diarrhea. It was the first canonical ligand identified that binds to and activates GUCY2C in animals and humans [7]. Media concentrations of cGMP were determined by EIA, and normalized to protein concentration of cell lysates of respective wells determined by BCA assay. Non-transfected CT26 cells were used as a negative control for ST treatment, an irrelevant mouse IgG was used as the control IgG.

### Immunotoxin Generation

IT conjugates were generated as previously described [37]. Briefly, cleavable sulfosuccinimidyl 6-[α-methyl-α-(2-pyridyldithio) toluamido] hexanoate (SMPT) or non-cleavable M-maleimid-obenzoyl-N-hydoxysuccinimide ester (MBS) activated the antibody through an amine reaction for 1 h at room temperature. Simultaneously, deglycosylated Ricin Toxin A (dgRTA) domain (Sigma-Aldrich) was reduced with DTT to insure free sulfhydryl groups. Activated antibodies and reduced dgRTA were desalted on C18 columns, and then reacted for 72-96 h at varying stoichiometric ratios. Immunotoxins were then purified by size exclusion chromatography and confirmed by gel electrophoresis.

### Immunotoxin ELISA

Binding competence of IT conjugates was confirmed using a GUCY2C extracellular domain (GUCY2C_1-430_)-based ELISA [25]. ITs were incubated in GUCY2C_1-430_-coated plates at varying concentrations. The mouse IgG component was detected with HRP-anti-mouse H + L (Jackson ImmunoResearch, #115-035-062). Ricin A chain was detected with rabbit-anti-Ricin antibody (Abcam, ab27169) followed by HRP-anti-rabbit H+L (Jackson ImmunoResearch, #111-035-003). Color was developed with Turbo-TMB substrate (Thermo Scientific, #34022) and quantified at λ480nm.

### Cell Imaging

Live imaging of sub-confluent cells plated on glass coverslips in 24 well plates was performed in media containing 25 mM HEPES. Prior to fixation, cells were incubated sequentially with primary antibody and fluorescent secondary antibody, and mounted on slides with DAPI Pro-long anti-fade mounting media and imaged by EVOS FL Auto (Life Technologies) or confocal microscopy (Zeiss 510M and Nikon C1 Plus, Thomas Jefferson University Bioimaging Shared Resource). Whole cell imaging was performed on CT26.GUCY2C cells, with parental CT26 cells as controls [23-25]. Methanol-fixed cells were co-stained with antibodies for lysosomal associated membrane protein 1 (LAMP1, Abcam #24170), clathrin and GUCY2C mAb and reviewed by confocal microscopy. Endocytosis was examined using thermomechanical control, in which internalization was prevented at temperatures <4°C [33]. Cell surface imaging was performed on live cells by sequential exposure to primary antibody and fluorescent secondary antibody at 4°C followed by fixation. GUCY2C internalization was evaluated in live cells by confocal microscopy by sequential exposure to primary and secondary antibodies at 4°C, followed by warming to room temperature while time-lapse imaging. Live cells for time-lapse were counter-stained with live cell-permeable markers Hoechst for nuclei, and lysotracker red for lysosomes.

### Targeted Inhibition of Gene Expression

GIPZ lentiviral mouse shRNA control or clathrin heavy chain constructs (Thermo Scientific) were transduced into sub-confluent cells by spinoculation for 1.5 h at 2700 RPM with VSV-G pseudo-typed lentivirus, and clathrin protein expression quantified at 48 h. Mouse siRNA scrambled control or siRNAs to caveolin (Cav-1; Ambion) were transfected into sub-confluent cells by using lipofectamine and Cav-1 protein expression quantified at 48 h.

### Immunotoxin Toxicity

Cells, plated in 96 well plates at 80 percent confluence one day prior to treatment, were exposed to various concentrations of ITs for 48 h. Cells were then exposed to MTT ((3-(4,5-Dimethylthiazol-2-yl)-2,5-diphenyltetrazolium bromide) for 1 h and then solubilized overnight at 37°C. Absorbance at 590 nm was measured for each well and percent cell kill quantified by comparison to non-treated wells (0% cell kill) and wells incubated with a concentration of cycloheximide which produced 100% cytotoxicity, as follows:

$$c\text{ toxicity}=\left(\frac{\text{experimental​formazon formed - vehicle formazan formed}}{\text{cvclohexamide formazan formed - vehicle formazan formed}}\right)x100$$

### Metastatic Tumor Model

Mice were inoculated with 5 × 10^5^ CT26.GUCY2C cells by tail vein 24 h prior to administration of 40 mg/kg of IT, or matched concentrations of mixed free antibody and toxin, every other day for 6 doses. Mice were sacrificed on day 13, lungs injected with India ink and fixed for 48 h, and tumors quantified by blinded enumeration.

### Tissue Histopathology

Hematoxylin and eosin-stained sections of the following organs were evaluated histologically by a board-certified veterinary pathologist (LDBB): brain, colon, epididymis, heart, kidney, liver, lung, salivary gland, small intestine, and spleen. Sections were specifically evaluated for histopathologic evidence of cell degeneration, necrosis, apoptosis, inflammation, vasculitis, atrophy, and regeneration. The percentage of tissue parenchyma with evidence of histopathologic lesions were scored as follows: None (0% of the parenchyma affected): 0; Rare (<1% of the parenchyma affected): 1; Occasional (1-5% of the parenchyma affected): 2; Minimal (6-10% of the parenchyma affected): 3; Mild (11-30% of the parenchyma affected): 4; Moderate (31-60% of the parenchyma affected): 5; and Marked (61-100% of the parenchyma affected): 6. Tissues from at least 3 mice per group were evaluated to compile scores.

### Statistical analysis

Statistical analyses were conducted using GraphPad Prism Software v5.

## SUPPLEMENTARY, TABLE AND FIGURE







## Abbreviations

Cav-1: caveolin-1
cGMP: cyclic GMP
DTT: dithiothreitol
EIA: enzyme-linked immunoassay
IF: immunofluorescence staining
IT: immunotoxin
GUCY2C: guanylyl cyclase c
HRP: horseradish peroxidase
LAMP1: lysosomal associated membrane protein 1
mAb: monoclonal antibody
RTA: Ricin Toxin A chain
ST: bacterial heat-stable enterotoxin
