# Supplementary material for: GUCY2C lysosomotropic endocytosis delivers immunotoxin therapy to metastatic colorectal cancer
Source: Oncotarget. 2014 Sep 8;5(19):9460–71. doi: 10.18632/oncotarget.2455 (PMC4253446; doi:10.18632/oncotarget.2455)
Supplement: Supplementary file 3 [file oncotarget-05-9460-s003.pdf]

GUCY2C lysosomotropic endocytosis delivers immunotoxin therapy to metastatic colorectal cancer

Supplementary Material

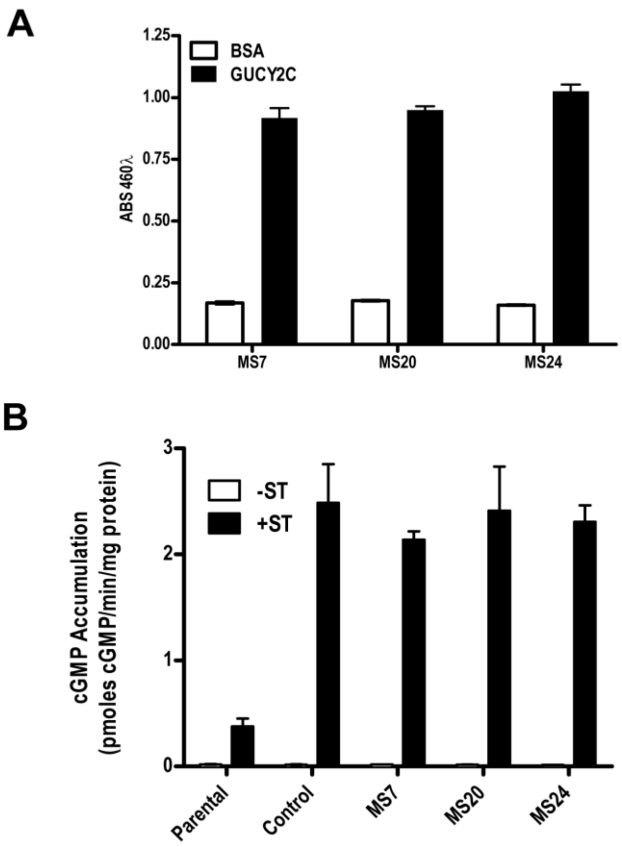

Supplemental Figure 1: GUCY2CmAb binds to, but does not activate, GUCY2C. (A) Plates coated with BSA or $GUCY2C_{1\text{-}430}$ were exposed to GUCY2CmAb (5 µg/mL) and developed with anti-mouse HRP. (B) Activation of GUYC2C cGMP production by its cognate ligand, the heat-stable enterotoxin (ST; 1 µM), in the presence or absence of GUCY2CmAb in CT26 or CT26.GUCY2C cells. Results are the mean ± SD of at least 3 determinations.

Supplemental Video 1: Three dimensional localization of GUCY2C in lysosomes. 200x magnification z-stacked with sub 1 micron sections of GUCY2C (green) and Lamp1 (red) in a mouse colonocyte demonstrating that co-localization (yellow) is preserved in all aspects of rotation rather than reflecting superimposed, but separate, subcellular localization.

Supplemental Video 2: Dynamic lysosomotropic endocytosis of GUCY2C in live cells. CT26.GUCY2C cells were labeled with lysotracker red (red), Hoechst for nuclear stain (blue), and MS20 (green) for 2 h at 4°C and then warmed to room temperature over 70 min and subjected to time-lapse imaging. Dynamic endocytosis of GUCY2C from the cell surface into lysosomes over time can be visualized by co-localization with lysotracker red (yellow fluorescence).

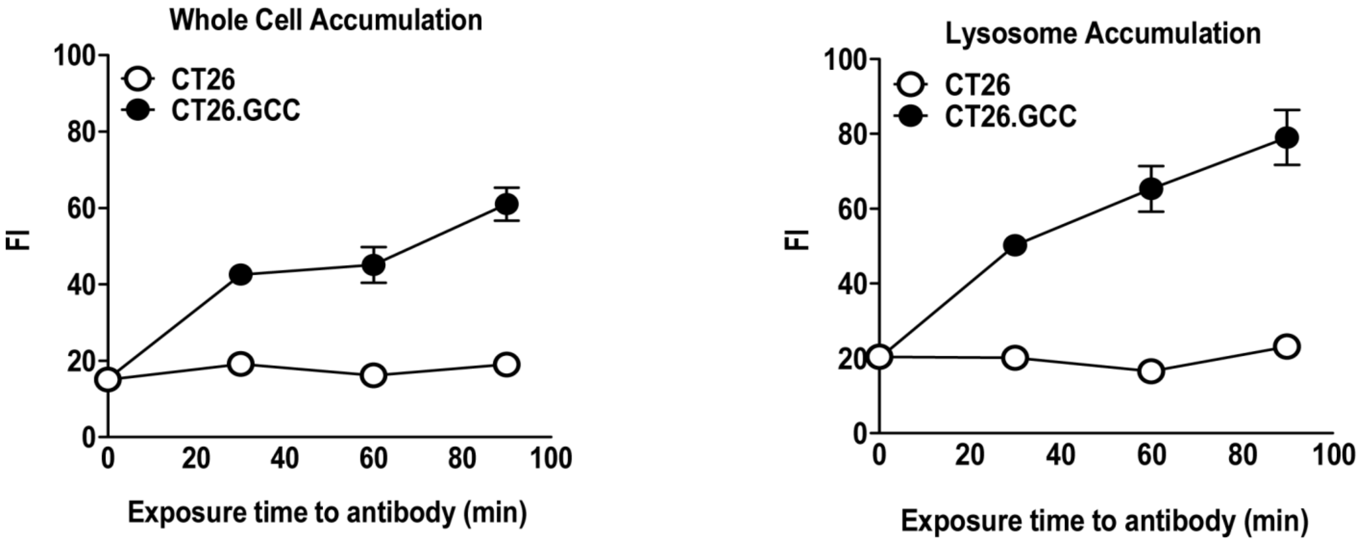

Supplemental Figure 2: Cell surface GUCY2C rapidly undergoes lysosomotropic endocytosis. CT26 or CT26.GCC cells were incubated with GUCY2CmAb at room temperature. At the indicated time points, cells were fixed with methanol and stained to detect GUCY2CmAb or lysotracker red. Fluorescence intensity was quantified with Image J in the entire intracellular compartment (left) or in lysosomes (right). Data represent the mean ± SD from at least 10 cells.

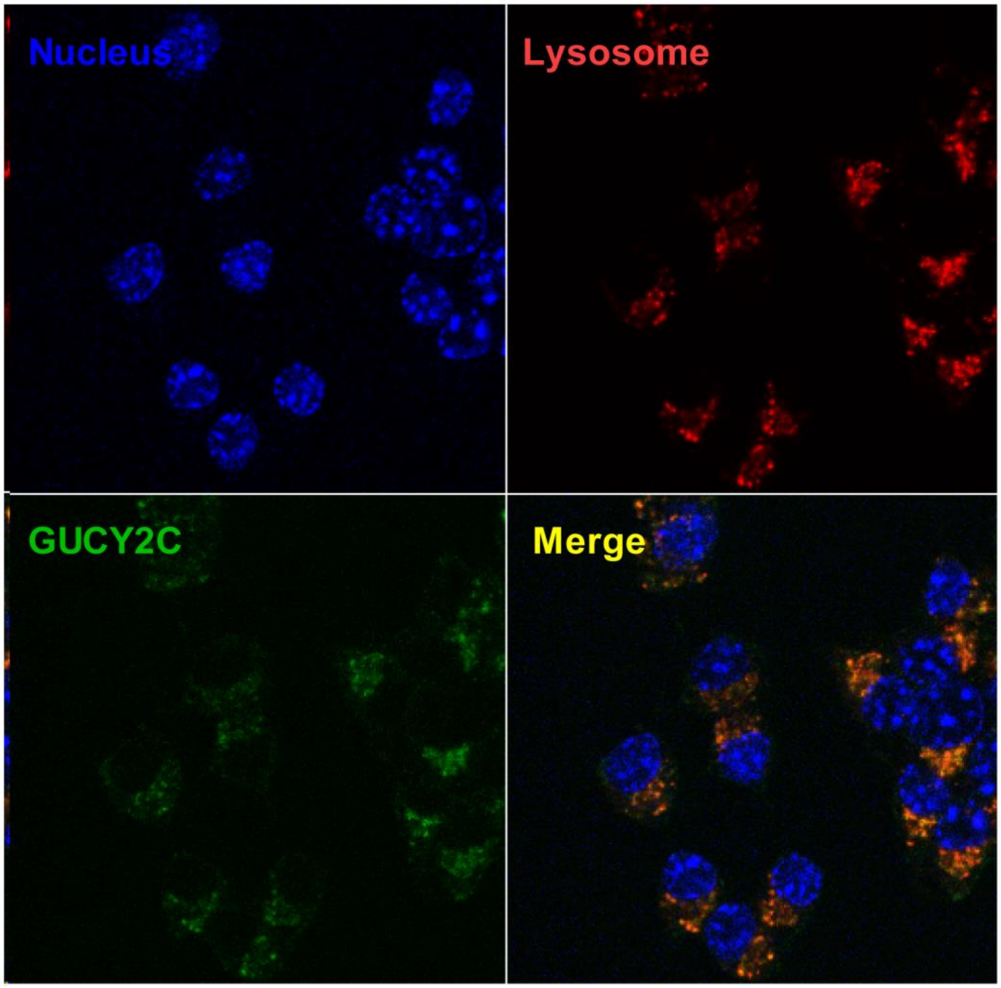

Supplemental Figure 3: GUCY2C endogenously expressed by colon cancer cells undergoes lysosomotropic endocytosis. (A) Immunofluorescent staining of live STC-1 murine intestinal cancer cells exposed to GUCY2CmAb (green) at 37°C for 5 h, washed and fixed and counter stained with DAPI for nuclei (blue) and lamp1 for lysosomes (red). Results are representative of at least 3 independent determinations.

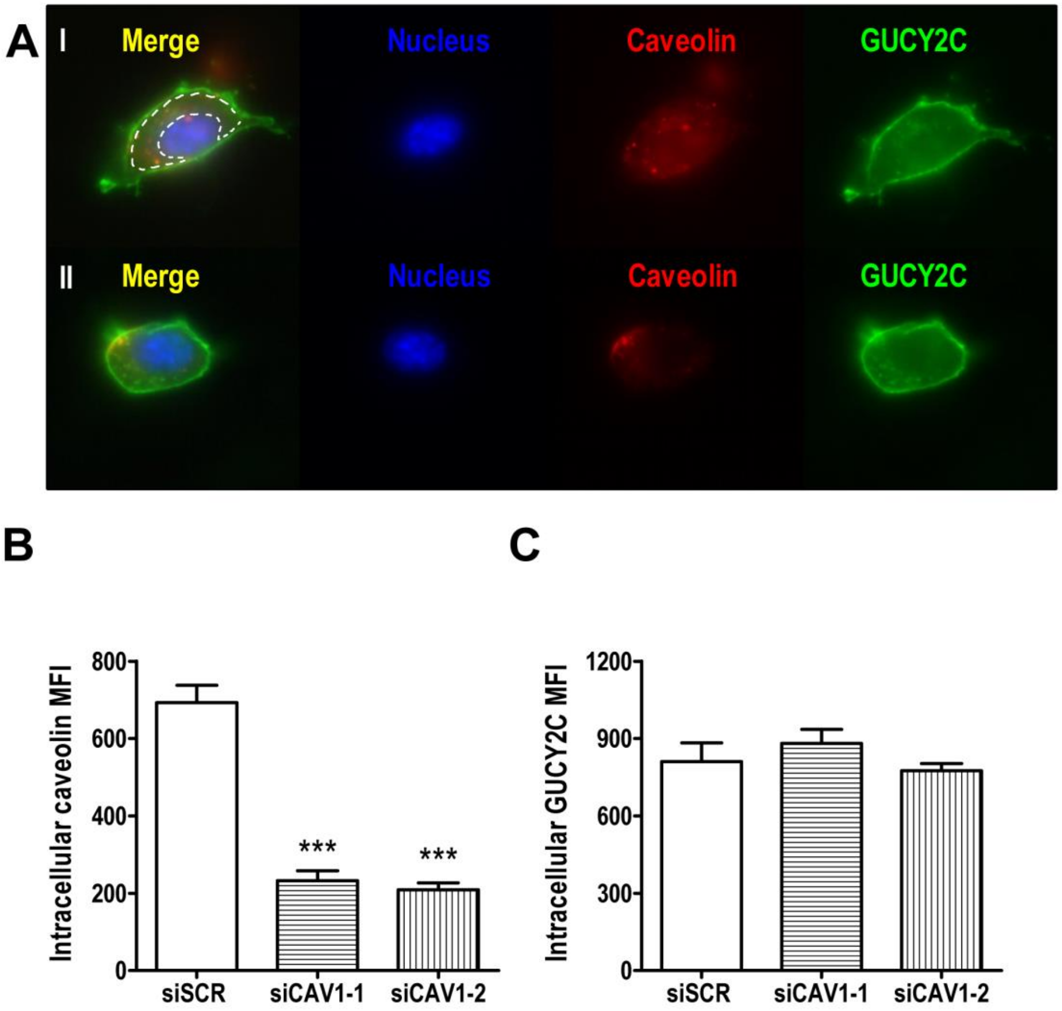

Supplemental Figure 4: Lysosomotropic endocytosis of GUCY2C is independent of caveolin. Representative single cell images showing internalized GUCY2C (green) detected as in A-C in cells with stably transfected control (I) or caveolin-specific (II) shRNA. Cells were counterstained with antibodies to clathrin (red) and DAPI for nuclei (blue). The intracellular compartment was selected for quantification (dotted white lines). (B) Quantification of caveolin knockdown and (C) GUCY2C internalization in cells treated with control (shCTRL) or two different caveolin shRNAs (shCAV-1, and 2). Analyses were performed in a blinded fashion and data are mean ± SEM of at least ten cells.

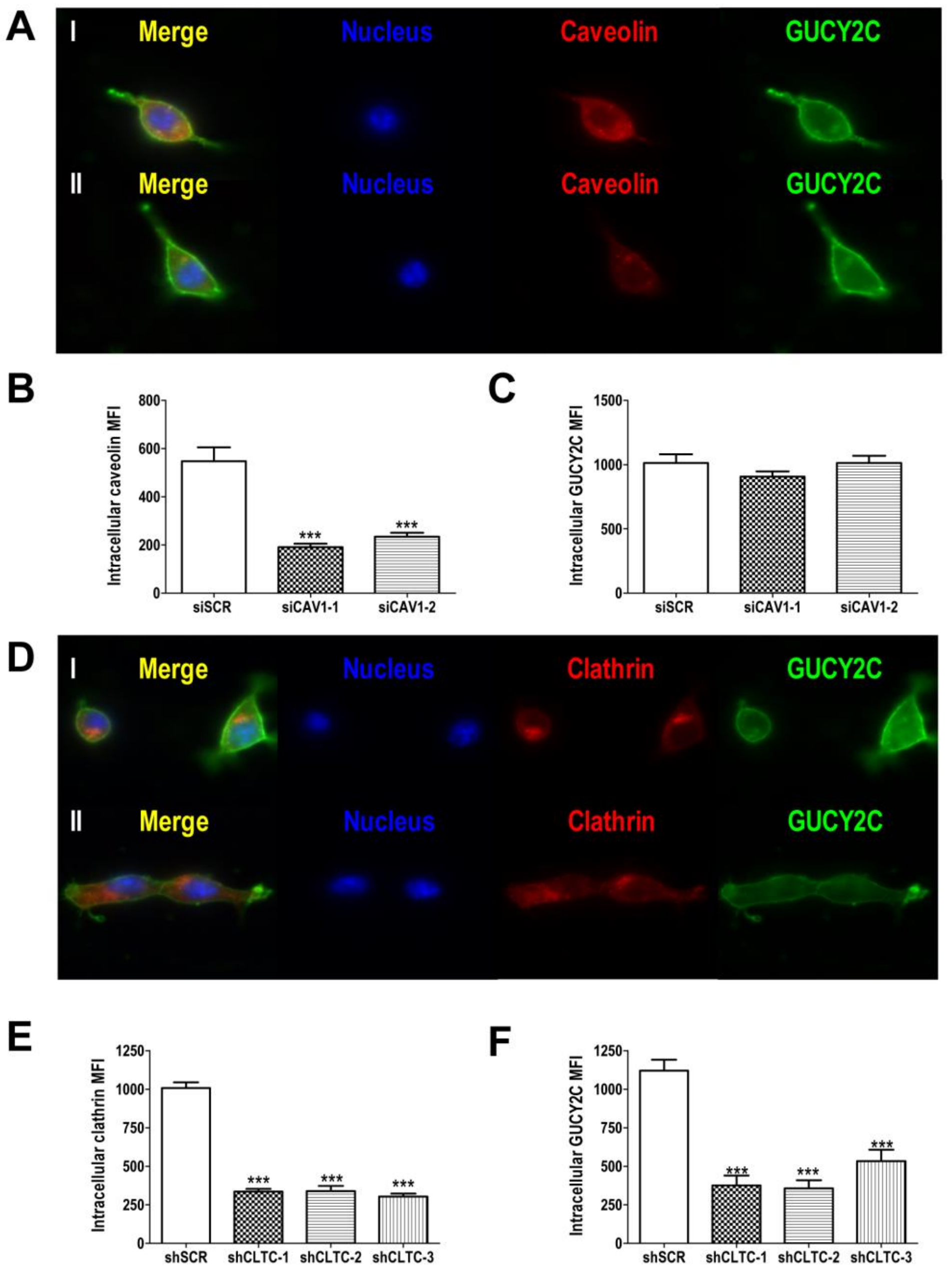

Supplemental Figure 5: Lysosomotropic endocytosis of GUCY2C is independent of the intracellular domain. CT26 cells stably expressing a GUCY2C truncation mutant lacking intracellular domains (CT26.GUCY2Ctm) were transfected with matched control (AI and DI), caveolin-specific (AII), or clathrin-specific (DII) shRNAs. Knockdown and GUCY2Ctm internalization were quantified as in Fig. 3. Data represent means ± SD of at least ten cells.

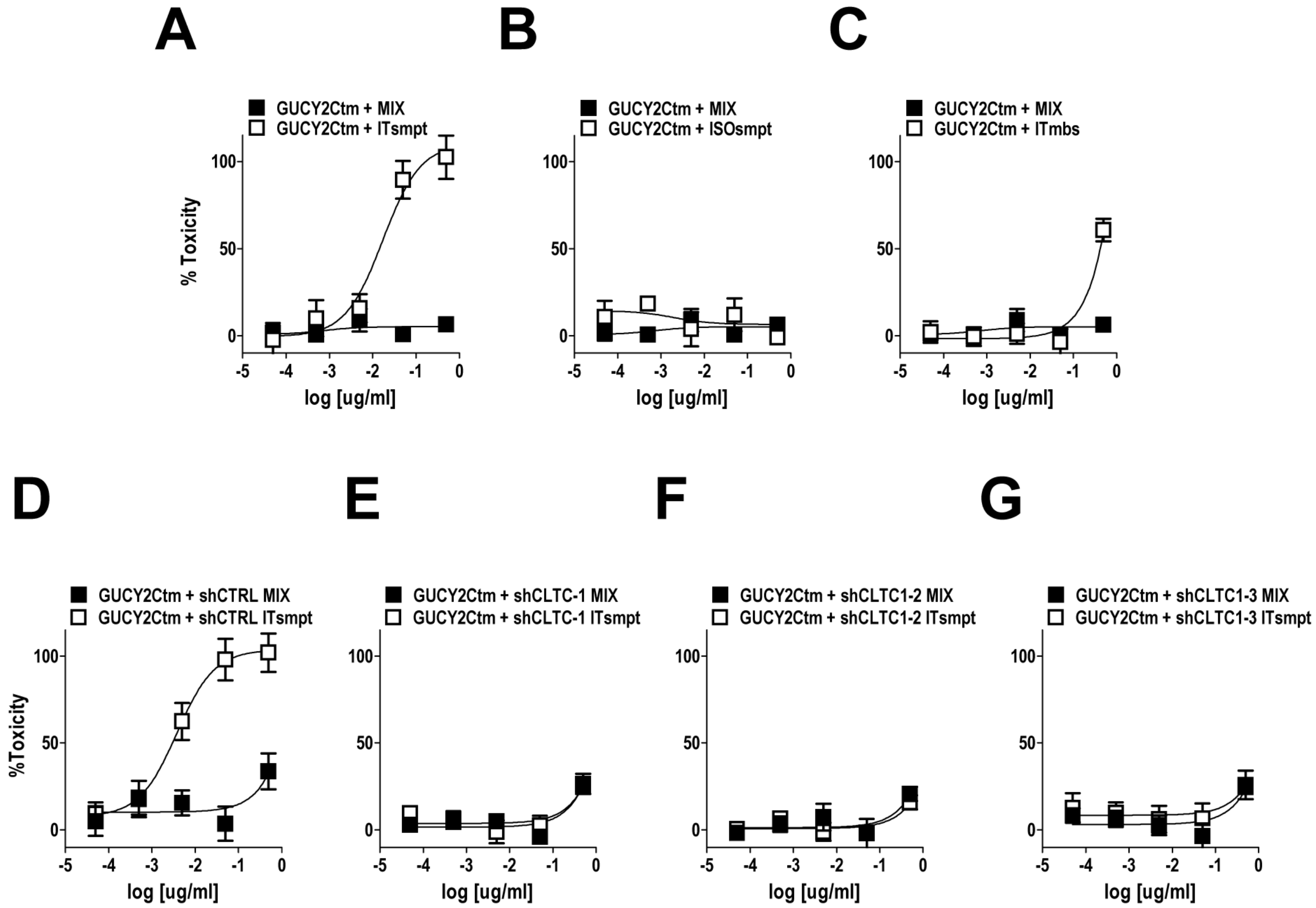

Supplemental Figure 6: IT cytotoxicity is independent of the GUCY2C intracellular domain. ITsmtp (A), ISOsmtp (B) and ITmbs (C) cytotoxicity was quantified in CT26.GUCY2Ctm, as outlined in Fig. 5. The clathrin-dependence of ITsmpt toxicity was assessed in CT26.GUCY2Ctm cells treated with (D) control or (E-G) clathrin-specific shRNAs, as outlined in Fig. 5. Results are the mean ± SEM of at least 3 independent determinations.

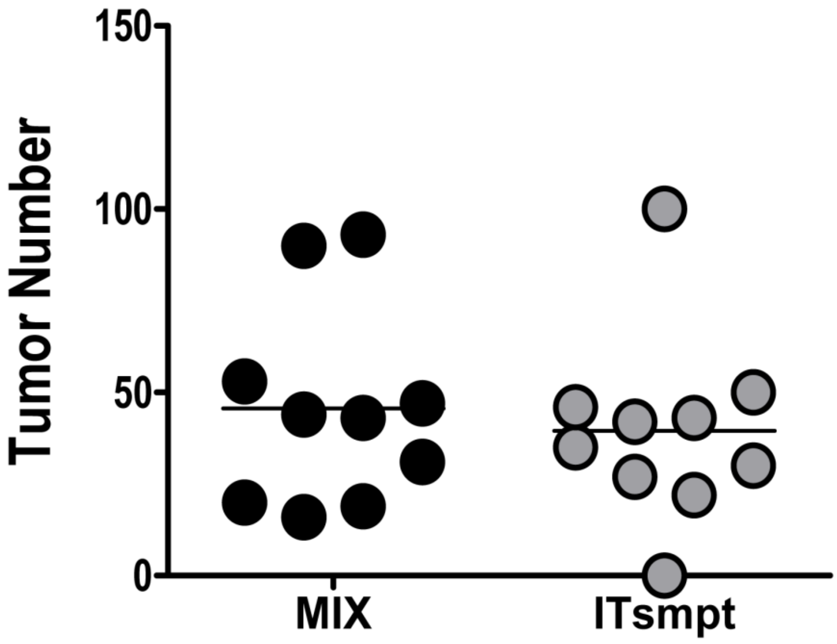

Supplemental Figure 7: GUCY2C-targeted ITsmpt is without effect on tumor burden in mice harboring GUCY2C-null colorectal cancer metastases in lung. Mice were challenged IV with 5 x 10^5 parental CT26 cells that are devoid of GUCY2C and treated with 0.4 mg/kg ITsmpt or MIX every other day beginning two days after challenge. Lungs were collected on day 14, stained to reveal tumors and enumerated. Results are representative of 10 mice per cohort.
